# Bio-Matrix Pot Addition Enhanced the Vegetation Process of Iron Tailings by *Pennisetum giganteum*

**DOI:** 10.3389/fmicb.2022.825660

**Published:** 2022-04-07

**Authors:** Yihao Liu, Jinyang Yu, Zuyu Wang, Petri Penttinen, Xiumei Yu, Ke Zhao, Menggen Ma, Quanju Xiang, Yunfu Gu, Hanjun Liu, Xiaoping Zhang, Qiang Chen

**Affiliations:** ^1^College of Resources, Sichuan Agricultural University, Chengdu, China; ^2^Safety and Environmental Protection Quality Supervision and Testing Research Institute, CNPC Chuanqing Drilling Engineering Co., Ltd., Guanghan, China

**Keywords:** iron tailing sands, bio-matrix pot, microbial communities, C and N cycle, vegetation

## Abstract

The barrenness of large mine tailing sand reservoirs increases the risks for landslides and erosion that may be accompanied with transfer of contaminants into the surrounding environment. The tailing sand is poor in nutrients, which effectively complicates the vegetation process. We investigated direct planting of *Pennisetum giganteum* into tailing sand using two pit planting methods: the plants were transplanted either directly into pits filled with soil or into soil-filled bio-matrix pots made of organic material. After growing *P. giganteum* in iron tailing sand for 360 days, the dry weight of the plants grown in the bio-matrix pot (T2) was approximately twofold higher than that of the plants grown in soil placed directly into the sand (T1). At 360 days, the organic matter (OM) content in the soil below the pit was the lowest in the not-planted treatment (T0) and the highest in T2, the available N (AN) contents were higher in T1 and T2 than in T0, and the available P and K contents were the highest in T2. At 360 days, the Shannon diversity of the soil microbial communities was higher in T1 and T2 than in T0, and the community compositions were clearly separated from each other. The profiles of predicted C cycle catabolism functions and N fixation-related functions in T1 and T2 at 360 days were different from those in the other communities. The results showed that *P. giganteum* grew well in the iron tailing sand, especially in the bio-matrix pot treatment, and the increased nutrient contents and changes in microbial communities indicated that using the bio-matrix pot in planting had potential to improve the vegetation process in iron tailing sands effectively.

## Introduction

The waste residue released from ore in mines after refining in the beneficiation plant is referred to as tailing sand (Dudeney et al., 2012; Kossoff et al., 2014; Lei et al., 2016). More than 10 billion tons of tailings are produced each year in the world, and approximately five billion tons of tailing sand are piled in China, with an annual increase of almost 500 million tons (Wang et al., 2017). The large mine tailing sand reservoirs are barren with no plants. The barrenness increases the risks for landslides and erosion that may be accompanied with transfer of contaminants into the surrounding environment. Tailing sand pond accidents, such as dam breaks and landslides, have been frequent and resulted in serious mudslides and collapses in recent years (Sun et al., 2018).

Several methods have been used to treat tailing sands, including refurbishment and reuse as building or chemical materials. However, the techniques are commonly of high cost and complicated or have resulted in secondary pollution (Alfonso et al., 2018; Galvão et al., 2018; Wu et al., 2018). Alarmingly, the wind-eroded dust from barren tailing sand reservoirs is a potent source of environmental contamination. In Fe-Pb-Zn mining area tailings in Murcia, Spain, the heavy metal concentrations in wind-eroded particulate matter were high, even though the concentrations in tailings were below hazardous levels (Khademi et al., 2018). Thus, the tailings should be stabilized. The tailings may be stabilized using chemical, for example, cementation, or biological methods, i.e., by vegetating the tailings. Tailing sand is poor in nutrients, which effectively complicates the vegetation process. Ensuring the growth of plants may require covering the tailings with soil, making the process laborious.

*Pennisetum giganteum* is a perennial gramineous plant that grows in the tropical, subtropical, and temperate area, with the merits of easy cultivation, drought and salinity tolerance, and the ability to thrive in barren soils (Hayat et al., 2020; Li et al., 2020). Since *P. giganteum* cultivation increased soil fertility and organic matter (OM) content in barren slopes or sandy decertified soils, it was often chosen as a pioneer plant for comprehensive ecological management in a variety of ecologically fragile areas (Hayat et al., 2020; Li et al., 2020).

In a preliminary study, a corn-based mini bio-matrix pot increased the growth of crop roots, yield, and microbial diversity in rhizosphere (unpublished). Thus, we hypothesized that in vegetating tailing sands, the bio-matrix pot would promote the growth of the plants and increase the OM and nutrient contents and microbial diversity in the sand. To test this, we applied two pit planting methods: the plants were transplanted either directly into pits filled with soil or into soil-filled bio-matrix pots; plant growth was measured, and soil properties and microbial communities as proxies of cultivability were analyzed.

## Materials and Methods

### Materials

Iron tailing sand was kindly provided by Chengdu Leejun Industrial Co., Ltd., Sichuan, China. The pH of the iron tailing sand was 7.90, and the sand contained OM 3.45 g kg^–1^, total nitrogen 0.12 g kg^–1^, total phosphorus 8.92 g kg^–1^, total potassium 7.96 g kg^–1^, alkaline nitrogen (AN) 16.64 mg kg^–1^, available phosphorus (AP) 1.78 mg kg^–1^, and available potassium (AK) 50.13 mg kg^–1^. The contents of available iron and exchangeable magnesium were 0.19 and 47.20 mg kg^–1^, respectively. The contents of chromium, copper, cadmium, lead, manganese, and zinc were 12.3, 52.4, 0.12, 4, 807, and 109 mg kg^–1^, respectively, all below the National Standard for Iron tailing sands in China (GB/T 31288-2014).

Soil for planting was from Jiange County (E105°28′52″, N32°0′52″), Sichuan, China. The soil was dried at room temperature, ground, and sieved through 20 mesh. The soil pH was 7.21; OM content was 11.70 g kg^–1^; and AN, AP, and AK contents were 35.98, 7.96, and 128.63 mg kg^–1^, respectively.

Bio-matrix pots were made by mixing corn cob powder (55%) and corn stalk powder (45%), adjusting water content to 55–60%, followed by autoclaving and cooling to room temperature. The mixture was inoculated with *Ganoderma lucidum* SZ-01, placed into a flowerpot mold, and incubated at 25°C for 10 days. During the incubation, the fungal mycelia bound the material firmly. After the incubation, the pots were dried at 80°C. The pots were 30- and 25-cm wide at the top and bottom, respectively, 20-cm high, and 2-cm thick. The OM, AN, AP, and AK contents of the pots were 642, 12.7, 21.7, and 52.8 g kg^–1^, respectively.

Two-to-three leaved, 15–20-cm high *P. giganteum* seedlings were purchased from the local growers.

### Experimental Design and Sampling

**FIGURE 1:**
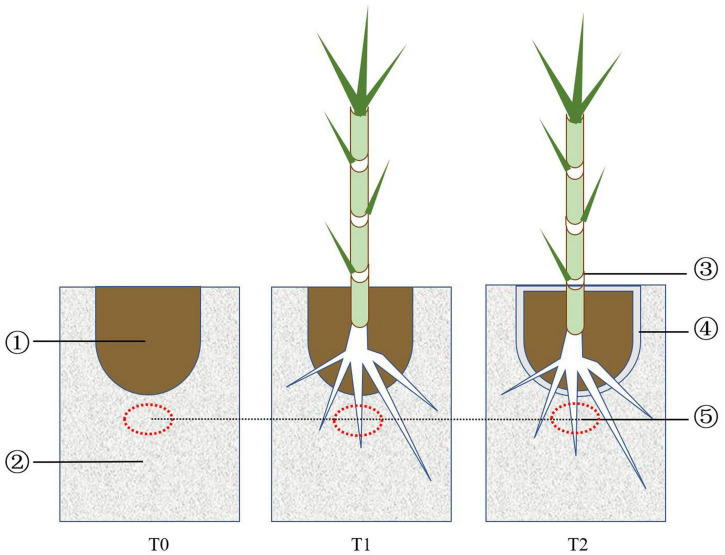
Design of the treatments: (1) soil, (2) iron tailing sand, (3) *Pennisetum giganteum* seedling, (4) bio-matrix pot, and (5) sampling spot.

The experiment, started in February 2016, was carried out in a greenhouse in 100 × 50 × 45 cm plastic containers. Three treatments were set up: iron tailing sand + soil (T0), iron tailing sand + soil + *P. giganteum* (T1), and iron tailing sand + soil + bio-matrix pot + *P. giganteum* (T2). Approximately 54 kg of iron tailing sand was placed into the container, and a hole the size of the bio-matrix pot was carved in the middle at the top (Figure 1). In T0 and T1, 6.5 kg of soil was placed into the hole; in T2, the soil was placed into the bio-matrix pot that was inserted into the hole. In T1 and T2, one *P. giganteum* seedling was transplanted into the soil. The containers were irrigated with distilled water.

Iron tailing sand samples from three containers per treatment were collected at 90, 180, and 360 days (Figure 1). The pH and OM, AN, AP, and AK contents were determined as described previously (Siddiqi and Glass, 1981). The plants were harvested at 360 days, and the shoot and root biomasses were measured after drying at 60°C to constant weight.

### 16S rRNA Gene Amplicon Sequencing

DNA was extracted from 0.5 g of sample using a Fast DNA ^®^ SPIN kit for soil (MP Biomedicals, United States) according to the manufacturer’s instructions. The DNA concentration and quality were assessed by 0.8% agarose gel electrophoresis and UV spectrophotometer (Thermo Scientific NC2000). DNA extracts were stored at −20°C.

The universal primers 338F (5′-ACTC CTA CGG GAG GCA GCA-3′) and 806R (5′-GGA CTA CHV GGG TWT CTA AT-3′) were used to amplify the V3–V4 region of the 16S rRNA gene of sample DNA (Huse et al., 2008; Göransson et al., 2011). The amplification was done in 25-μl reactions containing 5× reaction buffer 5 μl, 5× GC buffer 5 μl, dNTP (2.5 mM) 2 μl, forward primer (10 mM) 1 μl, reverse primer (10 mM) 1 μl, DNA template 2 μl, ddH_2_O 8.75 μl, and Q5 DNA Polymerase 0.25 μl (Magoc and Salzberg, 2011). The thermal program included denaturation at 98°C for 2 min, 26 cycles with denaturation at 98°C for 15 s, annealing at 55°C for 30 s, extension at 72°C for 30 s, and a final extension at 72°C for 5 min. The amplified products were separated by 2% agarose gel electrophoresis, and the target fragments were recovered using AXYGEN’s gel recovery kit (AXYGEN, United States). PCR products were mixed in equimolar ratios and purified using the GeneJET PCR Purification Kit (Thermo Fisher). Amplicon libraries were prepared using the NEBNext ^®^ Ultra™ DNA Library Prep Kit (New England Biolabs) prior to paired-end sequencing on the Illumina Miseq platform (Illumina, San Diego, CA, United States). The PCR products were sequenced at Shanghai Personal Medical Laboratory Co., Ltd. (Shanghai, China).

### Bioinformatics and Statistical Analysis

Illumina sequencing reads were paired and quality-filtered using the Quantitative Insights into Microbial Ecology (QIIME, v1.7.0) pipeline with default settings (Caporaso et al., 2010). Chimeric sequences were removed using UCHIME algorithm after alignment with the Gold database^1^ (Edgar, 2010). The sequences were assigned into operational taxonomic units (OTUs) at 97% similarity using UPARSE in the USEARCH pipeline v7.0.1090 (Edgar, 2010). The representative sequences of OTUs were assigned into taxa using SILVA rRNA database release 132 (Quast et al., 2013).

Rarefaction curves were created using R package vegan v2.5e2 in R studio (Oksanen et al., 2019). Chao1 and Shannon α-diversity indices at the OTU level were calculated in QIIME pipeline v1.7.0. The microbial communities were visualized at the genus level using cluster heatmaps based on 30 taxa with the highest relative abundances. The relative abundances of taxa were normalized using the *z*-score standardization method. The taxa were hierarchically clustered according to the Pearson correlation distance measure.

Microbial community composition at the genus level was visualized using principal component analysis (PCA) in R package vegan v2.5 in R studio (Oksanen et al., 2019). Partial least squares discriminant analysis (PLS-DA) at the OTU level was done using R packages BiocManager and mixOmics in R studio^2^ (Matthew and Rayens, 2003).

Differential abundance at the OTU level was analyzed pairwise at 90, 180, and 360 days using ALDEx2 (Fernandes et al., 2013). In the analysis, the underlying distributions were estimated using 128 Monte Carlo samples, the abundances were transformed using centered log-ratios, differences were tested using *t*-test, and effect sizes were measured as the relative fold difference. OTUs were considered differentially abundant when the effect size is >|1| and *p* < 0.1.

Carbon and nitrogen cycle-related functions were predicted using Functional Annotation of Prokaryotic Taxa (FAPROTAX) analysis in the Python-based FAPROTAX 1.1 package (Louca et al., 2016). Results were visualized as a clustered heatmap with a Pearson correlation dendrogram using the function “pheatmap” v1.0.12 in R studio.

Microsoft Excel 2016 was used to calculate means and standard deviations. Differences in plant and soil characteristics were tested using one-way ANOVA and Tukey’s test in IBM SPSS Statistics 20.0 (SPSS Inc., Chicago, IL, United States). Differences were taken as statistically significant at *p* < 0.05.

## Results

### Plant and Soil Characteristics

**FIGURE 2:**
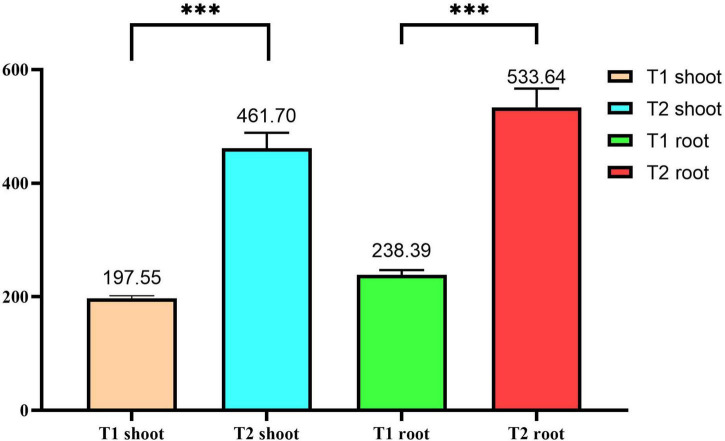
The shoot and root dry weights of *Pennisetum giganteum* grown in tailing sands for 360 days. The error bars indicate standard deviation (*n* = 3). ^***^Statistically significant difference (*p* < 0.05). T1, iron tailing sand + soil + *Pennisetum giganteum*; T2, iron tailing sand + soil + bio-matrix pot + *Pennisetum giganteum*.

After growing *P. giganteum* in iron tailing sand for 360 days, both the root dry weight (RDW) and shoot dry weight (SDW) of the plants grown in the bio-matrix pot (T2) were approximately twofold higher than those of the plants grown in soil placed directly into the sand (T1) (*p* < 0.05) (Figure 2), suggesting that the bio-matrix pot has promoted the growth of the plants.

The OM content of the iron tailing sand in T1 and T2 increased over time (Table 1). At 180 days, the OM content was the highest in T2, and at 360 days, the OM content was the lowest in the not-planted treatment (T0) and the highest in T2 (*p* < 0.05). The differences in pH values that ranged from 8.22 to 8.76 showed no clear trend. At 180 and 360 days, the AN contents were higher in T1 and T2 than in T0 (*p* < 0.05) (Table 1). The AP contents were the highest in T2 (*p* < 0.05). At 180 and 360 days, the AK contents were the highest in T2, and at 360 days, higher in T1 than in T0 (*p* < 0.05) (Table 1).

**TABLE 1:** The physicochemical properties of the iron tailing sand.

Physicochemical properties	T0^a^			T1			T2		
			
	90 d	180 d	360 d	90 d	180 d	360 d	90 d	180 d	360 d
OM (g kg^–1^)	3.78ab	3.62ab	3.24a	4.12ab	4.18ab	4.41b	3.72ab	5.33c	5.61c
pH	8.28a	8.59cd	8.53bc	8.75e	8.52bc	8.39ab	8.69de	8.71de	8.76e
Available N (mg kg^–1^)	22.41ab	16.67ab	12.39a	20.67ab	34.21cd	40.80d	26.36bc	35.14cd	43.07d
Available P (mg kg^–1^)	3.07a	3.27a	3.32a	2.80a	2.30a	3.60ab	5.73bc	6.75c	7.14c
Available K (mg kg^–1^)	60.50b	54.67ab	45.93a	59.53b	58.05b	61.75b	58.13b	77.00c	95.00d

*T0, iron tailing sand + soil; T1, iron tailing sand + soil + Pennisetum giganteum; T2, iron tailing sand + soil + bio-matrix pot + Pennisetum giganteum. 90 d, 180 d, and 360 d indicate the sampling time in days after the start of the experiment.*

*Different letters within the same row indicate statistically significant difference between treatments in individual sampling times tested by one-way ANOVA (p < 0.05).*

### Bacterial Communities

**FIGURE 3:**
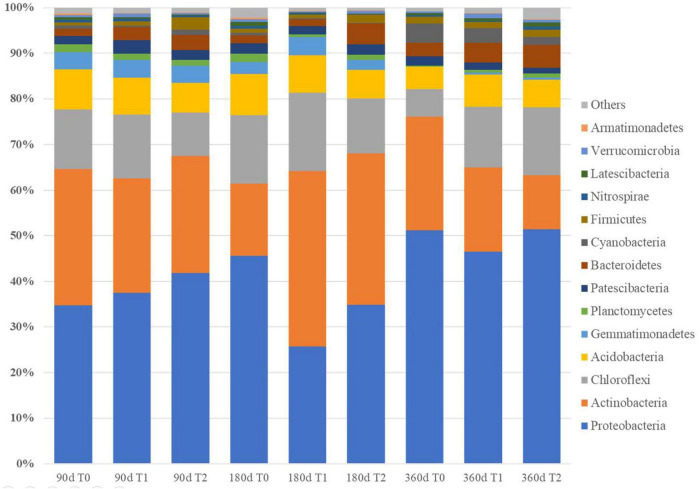
Bacterial community composition in the tailing sand at the phylum level. T0, iron tailing sand + soil; T1, iron tailing sand + soil + *Pennisetum giganteum*; T2, iron tailing sand + soil + bio-matrix pot + *Pennisetum giganteum*; 90 d, 180 d, and 360 d indicate the sampling time in days after the start of the experiment.

The 1,399,587 bacterial sequences from the 27 iron tailing sand samples were clustered into 1,436 OTUs at 97% similarity level. At 180 days, Chao1 index was higher in T1 than in T0 (*p* < 0.05) (Table 2). At 360 days, Shannon index was higher in T1 and T2 than in T0 (*p* < 0.05). Proteobacteria, Chloroflexi, Acidobacteria, and Actinobacteria were the most dominant phyla. The relative abundances of Proteobacteria accounted for 25.73–51.28% of the total abundance, Actinobacteria for 11.95–38.43%, Chloroflexi for 5.97–14.92%, and Acidobacteria for 4.92–8.76% (Figure 3 and Supplementary Table 1). At the genus level, the relative abundances of *Pseudomonas*, *Sideroxydans*, and *Geobacter* ranged from 0.53 to 9.41, 0.01 to 3.98, and 0.16 to 3.93%, respectively (Supplementary Table 2).

**TABLE 2:** The α-diversity of the microbial communities in the iron tailing sands.

α-Diversity	T0^a^			T1			T2		
			
	90 d	180 d	360 d	90 d	180 d	360 d	90 d	180 d	360 d
Simpson	0.9911ab	0.9914ab	0.9863a	0.9949ab	0.9916ab	0.9962b	0.9872ab	0.9963b	0.9967b
Chao1	2,794abcd	2,082ab	2,034a	3,458d	3,172cd	2,921abc	3,108bcd	2,317abcd	2,733abcd
ACE	3,005abc	2,216a	2,157a	3,654c	3,410bc	3,132abc	3,301abc	2,356ab	2,902abc
Shannon	9.68bc	9.29ab	8.73a	10.14c	9.82bc	10.09c	9.62bc	9.97bc	10.01c

*T0, iron tailing sand + soil; T1, iron tailing sand + soil + Pennisetum giganteum; T2, iron tailing sand + soil + bio-matrix pot + Pennisetum giganteum. 90 d, 180 d, and 360 d indicate the sampling time in days after the start of the experiment. Different letters within the same row indicate statistically significant difference between treatments in individual sampling times tested by one-way ANOVA (p < 0.05).*

**FIGURE 4:**
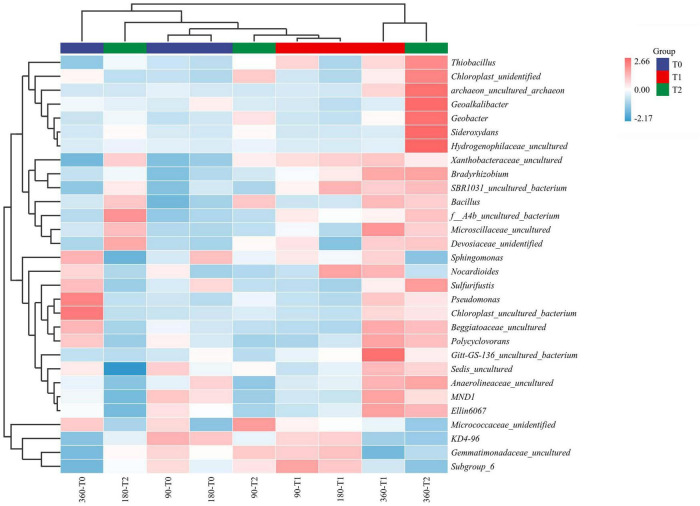
Bacterial community composition and their hierarchical clustering in the tailing sand at the genus level. T0, iron tailing sand + soil; T1, iron tailing sand + soil + *Pennisetum giganteum*; T2, iron tailing sand + soil + bio-matrix pot + *Pennisetum giganteum*; 90 d, 180 d, and 360 d indicate the sampling time in days after the start of the experiment.

**FIGURE 5:**
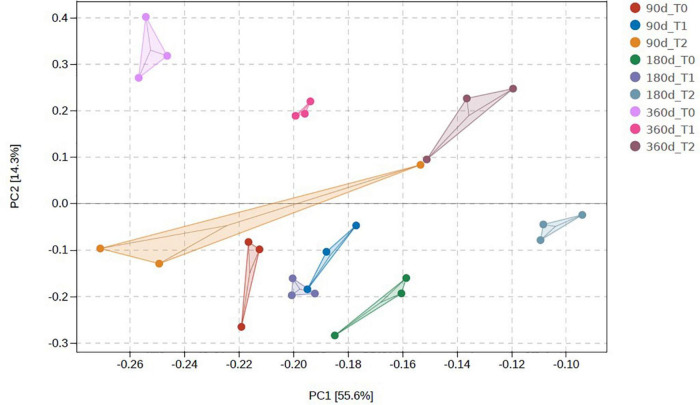
Principal component analysis of the microbial communities in the tailing sand at the operational taxonomic unit (OTU) level. T0, iron tailing sand + soil; T1, iron tailing sand + soil + *Pennisetum giganteum*; T2, iron tailing sand + soil + bio-matrix pot + *Pennisetum giganteum*; 90 d, 180 d, and 360 d indicate the sampling time in days after the start of the experiment.

**FIGURE 6:**
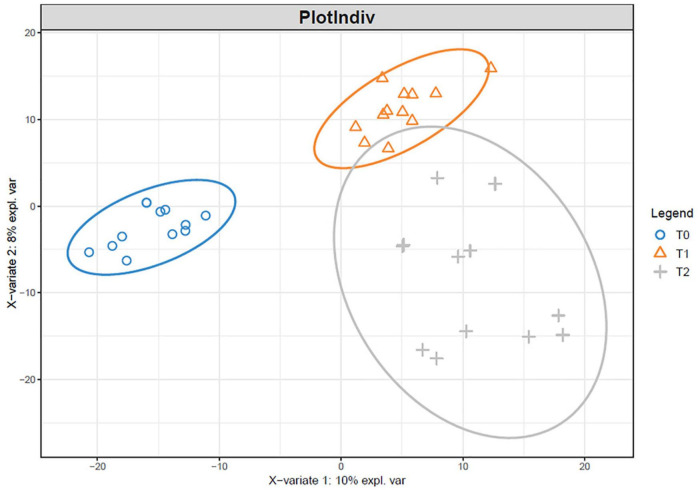
Partial least squares discriminant analysis of the microbial communities in the tailing sand at the OTU level. T0, iron tailing sand + soil; T1, iron tailing sand + soil + *Pennisetum giganteum*; T2, iron tailing sand + soil + bio-matrix pot + *Pennisetum giganteum*.

The microbial communities in T1 and T2 at 360 days were separated from the other communities in the hierarchical clustering (Figure 4). At 90 and 180 days, the communities in T0 and T1 were relatively similar in the PCA (Figure 5). The T2 community was separated from those in T0 and T1 at 180 days, and at 360 days, all the communities were clearly separated from each other (Figure 5). Based on the PLS-DA, the communities in T0 were different from those in T1 and T2 (Figure 6). At 180 days, the relative abundances of Anaerolineaceae (Chloroflexi) OTU7 and MND1 (Proteobacteria) OTU12 were lower in T2 than in T0 (effect size > |1|, *p* < 0.1) (Table 3). At 360 days, the relative abundances of Xanthobacteraceae (Proteobacteria) OTU24 and Marine Group II (Euryarchaeota) OTU59 were higher in T1 than in T0, and those of Micrococcaceae (Actinobacteria) OTU1, Solirubrobacteraceae (Actinobacteria) OTU30, *Rubellimicrobium* (Proteobacteria) OTU115, and Coleofasciculaceae (Cyanobacteria) OTU187 were lower (effect size > |1|, *p* < 0.1). The relative abundances of Sideroxydans (Proteobacteria) OTU31 and Marine Group II OTU59 were higher in T2 than in T0 at 360 days (effect size > |1|, *p* < 0.1). In the T1 treatment, the relative abundances of *Aeromicrobium* (Actinobacteria) OTU10 and Uncultured Gitt-GS-136 (Chloroflexi) OTU22 were higher at 180 and 360 days, respectively, than at 90 days (effect size > |1|, *p* < 0.1).

**TABLE 3:** Differentially abundant OTUs in the iron tailing sands.

	OTU	T0	T2	T0	T1	T2	Effect size
		180 d	180 d	360 d	360 d	360 d	
T1 vs T0	Unidentified *Micrococcaceae* (Actinobacteria) OTU1	181 ± 29	283 ± 64	**885 ± 85**	**494 ± 38**	213 ± 83	–5.1
	Uncultured *Xanthobacteraceae* (Proteobacteria) OTU24	49 ± 23	123 ± 22	**34 ± 7**	**126 ± 9**	102 ± 14	4.6
	Uncultured *Solirubrobacteraceae* (Actinobacteria) OTU30	30 ± 5	1 ± 2	**507 ± 247**	**10 ± 3**	7 ± 7	–5.8
	Uncultured Marine Group II (Euryarchaeota) OTU59	6 ± 4	2 ± 3	**4 ± 0**	**106 ± 10**	250 ± 63	6.1
	*Rubellimicrobium* (Proteobacteria) OTU115	22 ± 4	1 ± 1	**95 ± 17**	**7 ± 1**	2 ± 3	–7.0
	Uncultured *Coleofasciculaceae* (Cyanobacteria) OTU187	4 ± 3	0 ± 0	**100 ± 12**	**5 ± 2**	1 ± 1	–5.7
T2 vs T0	Uncultured *Anaerolineaceae* (Chloroflexi) OTU7	**304 ± 25**	**70 ± 14**	190 ± 27	351 ± 36	369 ± 192	–7.6
	MND1 (Proteobacteria) OTU12	**222 ± 16**	**50 ± 7**	162 ± 7	300 ± 8	214 ± 12	–7.7
	*Sideroxydans* (Proteobacteria) OTU31	2 ± 3	86 ± 72	**3 ± 1**	16 ± 0	**462 ± 111**	6.4
	Uncultured Marine Group II (Euryarchaeota) OTU59	6 ± 4	2 ± 3	**4 ± 0**	106 ± 10	**250 ± 63**	6.6

			***T1 90 d***	***T1 180 d***	***T1 360 d***		

180 vs 90 d	*Aeromicrobium* (Actinobacteria) OTU10		**112 ± 94**	**924 ± 81**	142 ± 8		9.0
360 vs 90 d	Uncultured Gitt-GS-136 (Chloroflexi) OTU22		**93 ± 14**	102 ± 59	**191 ± 2**		6.5

*The taxonomic identities of the OTUs are shown at the lowest identified taxonomic level; phyla are indicated in brackets. The relative abundances considered as differentially abundant in the ANOVA-like differential expression analysis (ALDEx2) are in bold. Effect sizes were measured as the relative fold difference of the differentially abundant OTUs between treatments.*

*T0, iron tailing sand + soil; T1, iron tailing sand + soil + Pennisetum giganteum; T2, iron tailing sand + soil + bio-matrix pot + Pennisetum giganteum. 90 d, 180 d, and 360 d indicate the sampling time in days after the start of the experiment. The relative abundances are as mean ± standard deviation (n = 3).*

### Predicted C and N Cycle-Related Functions

**FIGURE 7:**
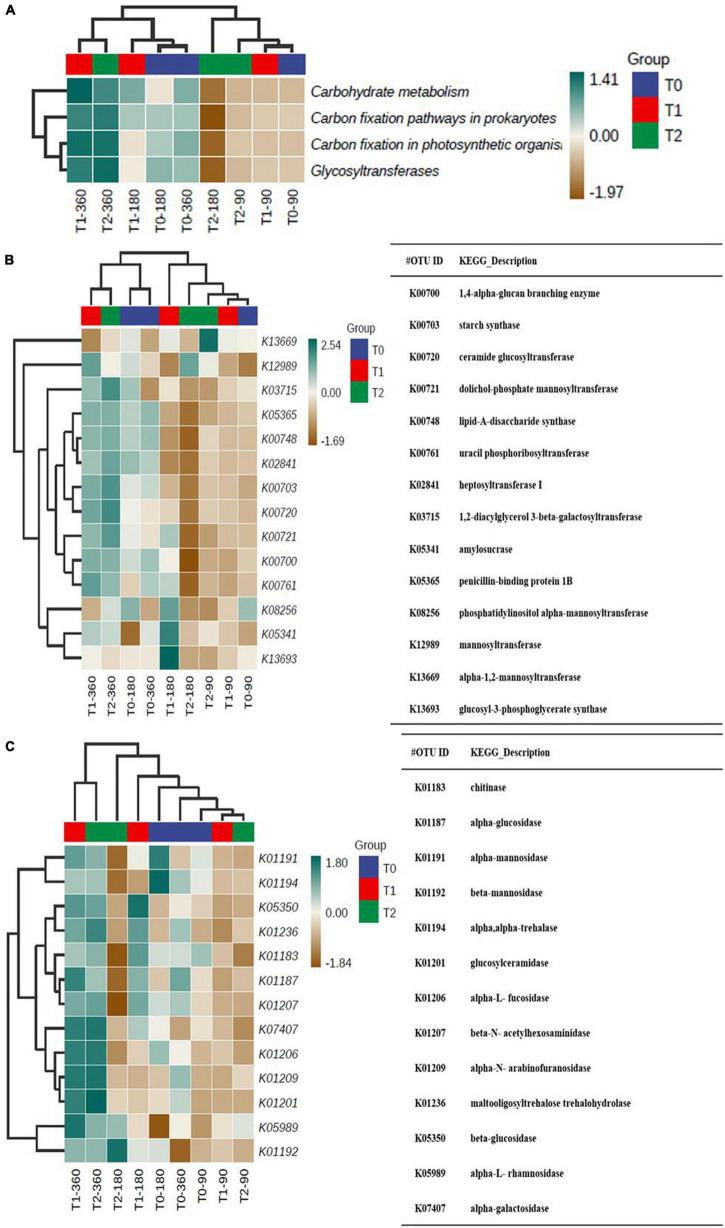
**(A)** The predicted C cycle-related functions in the microbial communities in the tailing sand samples and the predicted C cycle **(B)** biosynthesis and **(C)** catabolism profiles. T0, iron tailing sand + soil; T1, iron tailing sand + soil + *Pennisetum giganteum*; T2, iron tailing sand + soil + bio-matrix pot + *Pennisetum giganteum*. 90 d, 180 d, and 360 d indicate the sampling time in days after the start of the experiment.

We predicted C and N cycle-related functions to estimate the accompanying changes on gene level. In line with the OM content increase over time in T1 and T2, the predicted functions related to carbohydrate metabolism, C fixation in prokaryotes and photosynthetic organisms, and glycosyltransferases were on the same level at 90 days and high in T1 and T2 at 360 days (Figure 7A). The predicted C cycle biosynthesis and catabolism profiles were similar at 90 days (Figures 7B,C). The profiles of predicted biosynthesis functions in T1 and T2 at 360 days were relatively similar to those in T0 at 180 and 360 days (Figure 7B). The profiles of predicted C cycle catabolism functions in T1 and T2 at 360 days were different from those in the other communities (Figure 7C).

**FIGURE 8:**
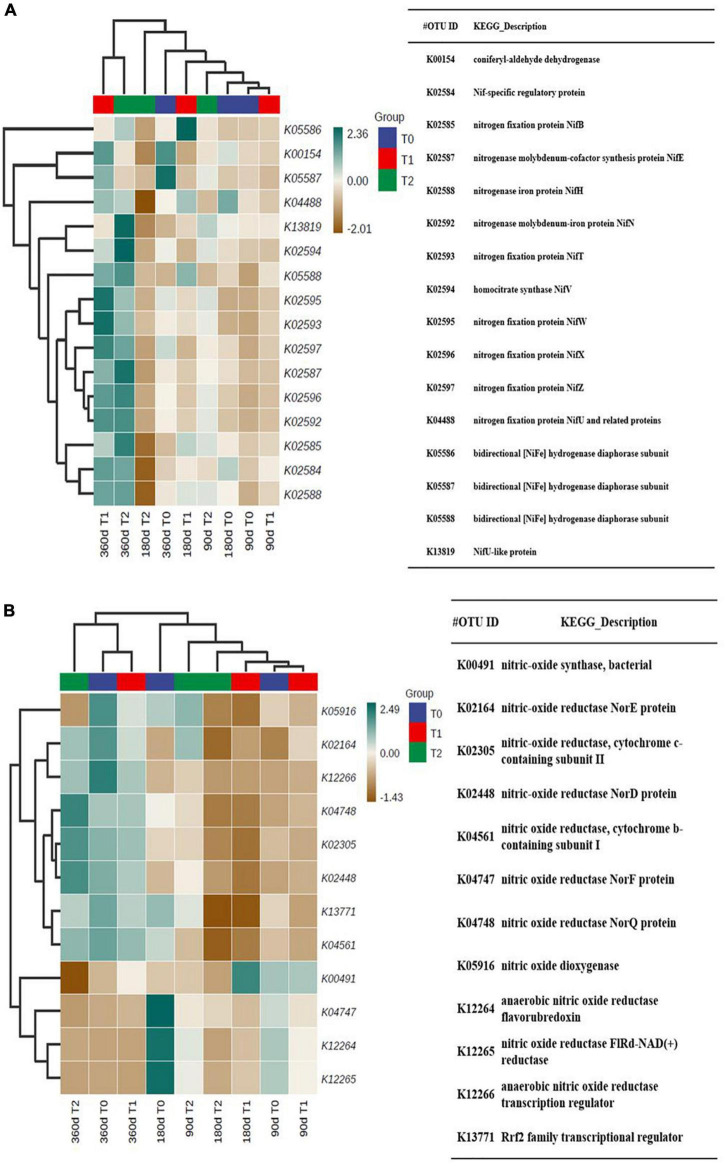
The predicted **(A)** N fixation-related functions and **(B)** denitrification-related functions in the microbial communities in the tailing sand samples. T0, iron tailing sand + soil; T1, iron tailing sand + soil + *Pennisetum giganteum*; T2, iron tailing sand + soil + bio-matrix pot + *Pennisetum giganteum*. 90 d, 180 d, and 360 d indicate the sampling time in days after the start of the experiment.

The predicted N fixation-related functions were on approximately the same level at 90 and 180 days and high in T1 and T2 at 360 days (Figure 8A). The predicted denitrification-related functions were on approximately the same level at 360 days (Figure 8B).

## Discussion

The large mine tailing sand reservoirs are barren with no plants, making them prone to landslides and erosion. Vegetating the tailings is recommended to prevent the transfer of contaminants into surrounding environment (Khademi et al., 2018), yet it is complicated due to the poor nutrient content of the tailing sand. We investigated direct planting into tailing sand using two pit planting methods: the plants were transplanted either directly into pits filled with soil or into soil-filled bio-matrix pots made of corn and *G. lucidum*.

The results showed that *P. giganteum* grew well in the iron tailing sand, especially in the bio-matrix pot treatment (T2). The higher biomass in T2 may have been due to the *G. lucidum* polysaccharides that are known to promote the growth of cotton (Zhang et al., 2019). The OM and AN contents increased in the planted iron tailing sand in the directly-into-soil-planted treatment (T1) and T2, whereas in the not-planted soil-only treatment (T0), the contents remained on the same level, indicating that the increases were due to the roots penetrating into the sand. At the end of the experiment, the AK had increased in the planted treatments and were the highest in T2. This, together with the over two times higher biomass of *P. giganteum* in T2 than in T1, indicated that using the bio-matrix pot in planting had potential to improve the vegetation process in iron tailing sands effectively.

The physicochemical properties of soil affect the diversity and activity of bacterial communities, and in natural tailing restoration, the diversity of soil microbial communities increases along the steps (Colin et al., 2019). In our study, at the end of the experiment, the diversity was higher in the planted treatments than in T0. As for the OM and AN content, the diversity increase was probably due to the plants and not only because of the soil in the pits. The community compositions were relatively similar at 90 days and in T0 and T1 even at 180 days, implying that the community composition changed at a faster rate in T2, possibly due to the quicker build-up of OM in T2. At the end of the experiment at 360 days, all the communities were clearly separated, implying that both the planting and the planting method had contributed to the differences in composition. Consistent with previous studies (Wang et al., 2017), Proteobacteria, Chloroflexi, Acidobacteria, and Actinobacteria were the dominant phyla, indicating the microbial communities have good adaptability. In barren gold mine tailings, Actinobacteria was the dominant phylum (Sibanda et al., 2019). In tailings undergoing restoration, Proteobacteria increased along the restoration process, and the abundance of actinobacteria decreased after remediation (Wu et al., 2018; Xu et al., 2018; Yu et al., 2019). In agreement, the differentially abundant OTUs were mostly assigned into Proteobacteria and Actinobacteria. Despite the community-level differences, the number of differentially abundant OTUs was small due to the large within-treatment variation.

The effect of the treatments on soil C and N cycle-related functions was estimated based on predicted functional profiles of microbial communities. At 360 days, functions related to C cycle catabolism and N fixation were estimated to be more abundant in the planted treatments, whereas the C cycle biosynthesis and denitrification-related functions seemed to change similarly in the treatments over time. Presumably, the increase in OM content with planting explained the increase in C cycle catabolism. In copper mine tailings, the diversity of nitrogen-fixing bacteria increased along natural restoration time together with development of pioneer plant communities (Zhan and Sun, 2012; Xiao et al., 2019). The increase in predicted N fixation functions implied that both planting methods had the potential to promote further development of vegetation, which is often limited by the availability of N (Göransson et al., 2011).

## Conclusion

The results showed that *P. giganteum* grew well in the iron tailing sand, especially in the bio-matrix pot treatment, and increased the nutrient contents, indicating that using the bio-matrix pot in planting had the potential to improve the vegetation process in iron tailing sands effectively. At the end of the experiment, the diversity of the microbial communities in the tailing sands was higher in the planted treatments. The increase in predicted N fixation functions implied that both planting methods had the potential to promote further development of vegetation.

## Data Availability Statement

The original contributions presented in the study are included in the article/Supplementary Material, further inquiries can be directed to the corresponding authors.

## Author Contributions

QC, XZ, and YL designed the research. YL, JY, and ZW performed the research. YL, YG, MM, KZ, QX, and HL analyzed the data. YL, QC, XZ, XY, and PP analyzed the data and wrote the manuscript. All authors contributed to the article and approved the submitted version.

## Conflict of Interest

HL was employed by the company CNPC Chuanqing Drilling Engineering Co., Ltd. The remaining authors declare that the research was conducted in the absence of any commercial or financial relationships that could be construed as a potential conflict of interest.

## Publisher’s Note

All claims expressed in this article are solely those of the authors and do not necessarily represent those of their affiliated organizations, or those of the publisher, the editors and the reviewers. Any product that may be evaluated in this article, or claim that may be made by its manufacturer, is not guaranteed or endorsed by the publisher.

## Footnotes

1
https://drive5.com/uchime/gold.fa

2
10.18129/B9.bioc.mixOmics
